# Stress Sensitivity, Aberrant Salience, and Threat Anticipation in Early Psychosis: An Experience Sampling Study

**DOI:** 10.1093/schbul/sbv190

**Published:** 2016-02-01

**Authors:** Ulrich Reininghaus, Matthew J. Kempton, Lucia Valmaggia, Tom K. J. Craig, Philippa Garety, Adanna Onyejiaka, Charlotte Gayer-Anderson, Suzanne H. So, Kathryn Hubbard, Stephanie Beards, Paola Dazzan, Carmine Pariante, Valeria Mondelli, Helen L. Fisher, John G. Mills, Wolfgang Viechtbauer, Philip McGuire, Jim van Os, Robin M. Murray, Til Wykes, Inez Myin-Germeys, Craig Morgan

**Affiliations:** ^1^Centre for Epidemiology and Public Health, Health Service and Population Research Department, Institute of Psychiatry, Psychology and Neuroscience, King’s College London, London, UK;; ^2^Department of Psychiatry and Neuropsychology, School for Mental Health and Neuroscience, Maastricht University, Maastricht, The Netherlands;; ^3^Psychosis Studies Department, Institute of Psychiatry, Psychology and Neuroscience, King’s College London, London, UK;; ^4^Psychology Department, Institute of Psychiatry, Psychology and Neuroscience, King’s College London, London, UK;; ^5^Department of Psychology, The Chinese University of Hong Kong, Hong Kong, China;; ^6^National Institute for Health Research (NIHR) Mental Health Biomedical Research Centre at South London and Maudsley NHS Foundation Trust and King’s College London, London, UK;; ^7^Department of Psychological Medicine, Institute of Psychiatry, Psychology and Neuroscience, King’s College London, London, UK;; ^8^MRC Social, Genetic & Developmental Psychiatry Centre, Institute of Psychiatry, Psychology and Neuroscience, King’s College London, London, UK;; ^9^Center for Contextual Psychiatry, Department of Neuroscience, Catholic University of Leuven, Leuven, Belgium

**Keywords:** stress sensitivity, aberrant salience, threat anticipation, ecological momentary assessment, prodrome, first-episode psychosis

## Abstract

While contemporary models of psychosis have proposed a number of putative psychological mechanisms, how these impact on individuals to increase intensity of psychotic experiences in real life, outside the research laboratory, remains unclear. We aimed to investigate whether elevated stress sensitivity, experiences of aberrant novelty and salience, and enhanced anticipation of threat contribute to the development of psychotic experiences in daily life. We used the experience sampling method (ESM) to assess stress, negative affect, aberrant salience, threat anticipation, and psychotic experiences in 51 individuals with first-episode psychosis (FEP), 46 individuals with an at-risk mental state (ARMS) for psychosis, and 53 controls with no personal or family history of psychosis. Linear mixed models were used to account for the multilevel structure of ESM data. In all 3 groups, elevated stress sensitivity, aberrant salience, and enhanced threat anticipation were associated with an increased intensity of psychotic experiences. However, elevated sensitivity to minor stressful events (χ^2^ = 6.3, *P* = 0.044), activities (χ^2^ = 6.7, *P* = 0.036), and areas (χ^2^ = 9.4, *P* = 0.009) and enhanced threat anticipation (χ^2^ = 9.3, *P* = 0.009) were associated with more intense psychotic experiences in FEP individuals than controls. Sensitivity to outsider status (χ^2^ = 5.7, *P* = 0.058) and aberrantly salient experiences (χ^2^ = 12.3, *P* = 0.002) were more strongly associated with psychotic experiences in ARMS individuals than controls. Our findings suggest that stress sensitivity, aberrant salience, and threat anticipation are important psychological processes in the development of psychotic experiences in daily life in the early stages of the disorder.

## Introduction

Subclinical psychotic experiences are common in the general population^1–3^ and associated with an increased probability of developing a psychotic disorder.^1^ This suggests that psychotic experiences may be phenomenologically and temporally continuous,^1^ extending from subclinical psychotic experiences to psychotic disorder.^1,4,5^ Contemporary models of psychosis have proposed several psychological mechanisms that may contribute across different phenomenological and temporal stages to the development of psychosis.^6–11^ Targeting these at an early stage is potentially useful for achieving better outcomes of psychosis,^12–18^ but our understanding of how psychological mechanisms impact at different stages on, and increase the intensity of, psychotic experiences in individuals’ daily lives, outside the research laboratory^19,20^ remains limited.

To date, the psychological mechanism most widely studied in daily life is elevated stress sensitivity, characterized by intense emotional reactions to minor stressors and routine daily hassles.^20,21^ Previous research suggests emotional reactivity to minor stressful events, activities, and social situations is increased in individuals with enduring psychosis and in those with higher familial or psychometric risk.^20–24^ One study of individuals with an at-risk mental state (ARMS), also known as high-risk or ultra-high-risk state,^5,25^ reported greater emotional reactivity to minor activity-related and social stress in this group.^26^ However, no study has investigated the role of stress sensitivity in individuals with first-episode psychosis (FEP). This would allow us to minimize the effects of illness chronicity and further elucidate the impact of this mechanism across different stages of psychosis. Also, while there is evidence that minor stressors are associated with psychotic experiences,^20–22,26,27^ no study has specifically tested whether elevated stress *sensitivity* (ie, more *intense emotional reactions to* minor stressors) per se contributes to the development of more intense psychotic experiences in daily life.

While recent reviews suggest several socio-environmental factors are associated with psychosis (eg, urbanicity, ethnic minority status),^10,11,28,29^ the psychological mechanisms underlying an individuals’ subjective experience of these factors in daily life are poorly understood. Some individuals may experience stronger emotional reactions to unpleasant neighbourhoods and, thereby, develop more intense psychotic experiences. Further, exposure to socio-environmental factors (eg, social disadvantage,^30–33^ ethnic minority status^10,11^) may sensitize, and increase emotional reactivity of, individuals to subjective experiences of outsider status, and so increase intensity of psychotic experiences in daily life.^10,34^

It has been further suggested that exposure to social adversity sensitizes the mesolimbic dopaminergic system.^9–11,35^ Kapur^36^ proposes that excess striatal dopamine may lead to aberrant assignment of salience to otherwise irrelevant stimuli.^9,35–39^ According to this model, psychotic experiences emerge as a “top-down” cognitive attempt to make sense of experiences that are aberrantly salient.^36^ While there is some evidence on this model from experimental tasks,^40–42^ evidence on individuals’ subjective experience of aberrant salience, which may be particularly relevant to subclinical and attenuated psychotic experiences,^40,41,43^ remains limited.

Another putative psychological mechanism underlying psychotic experiences is enhanced anticipation of threat.^14,44–47^ Repeated exposure to adversity may lead individuals to anticipate more unpleasant events from their environment to create an enduring sense of threat anticipation.^10,44,45^ Bentall et al^44,45^ argued that this mechanism may be particularly important in the final stage of developing clinical psychosis, but this has yet to be tested in the daily lives of individuals with psychotic disorder in comparison to ARMS individuals and controls.

Our overall aim was to investigate whether elevated stress sensitivity, aberrant salience, and threat anticipation are important mechanisms in the development of psychotic experiences in daily life. To this end, we used the experience sampling method (ESM), a structured, random time-sampling diary technique, in a sample of individuals with FEP, individuals with ARMS, and controls to test the following hypotheses: (1) within each group (FEP, ARMS, controls), elevated stress sensitivity, experiences of aberrant salience, and enhanced threat anticipation are associated with an increased intensity of psychotic experiences; and (2) these associations are stronger in FEP and ARMS individuals than in controls.

## Method

### Sample

We recruited a sample of FEP individuals, ARMS individuals, and controls identified in the Childhood Adversity and Psychosis study and “The European Network of National Networks studying Gene-Environment Interactions in Schizophrenia” (EU-GEI),^6^ respectively.

*FEP*. FEP individuals were recruited from mental health services (MHS) in south-east London. Inclusion criteria were: aged 18–64; resident within defined catchment areas; presence of a FEP (ICD-10 F20–F29, F30–F33)^48^; adequate command of the English language. Exclusion criteria were: transient psychotic symptoms resulting from acute intoxication; psychotic symptoms precipitated by an organic cause; IQ<60, measured with an adapted version of the Wechsler Adult Intelligence Scale (WAIS).^6,49^ For participants in hospital at time of consent, ESM assessments were completed when they were discharged.

*ARMS.* ARMS individuals were recruited from Outreach and Support in South London (OASIS), a clinical service for people at high risk of psychosis provided by the South London and Maudsley NHS Foundation Trust,^50^ the West London Mental Health NHS Trust (WLMHT), and a community survey of general practitioner (GP) practices. Inclusion criteria were: aged 18–35, presence of an ARMS based on the comprehensive assessment of at-risk mental states (CAARMS)^5,6^ (supplementary table 1) or the Schizophrenia Proneness Instrument—Adult version (SPI-A) (ie, meeting the at-risk criterion of cognitive-perceptive basic symptoms),^51–55^ and adequate command of the English language. Exclusion criteria were: prior experience of a psychotic episode for more than 1 week as determined by the CAARMS and Structured Clinical Interview for DSM Disorders (SCID),^56^ previous treatment with an antipsychotic for a psychotic episode, and IQ <60 (measured as above).^6,49^

*Controls*. Controls were recruited using GP lists (including all registered patients for whom the practice is responsible for providing primary medical services) and the national postal address file as sampling frames. Inclusion criteria were: aged 18–64, resident within same areas as FEP individuals, and adequate command of the English language. Exclusion criteria for controls were the same as for FEP individuals with the addition of the following: personal/family history of psychotic disorder,^57^ presence of psychotic symptoms, measured with the Psychosis Screening Questionnaire (PSQ),^58^ presence of an ARMS based on the CAARMS or SPI-A (see above criteria), and IQ <60 (measured as above).^6,49^

All participants entered the study between June 2012 and August 2014. Full ethical approval for all aspects of the study was obtained from the National Research Ethics Service Committee London Central.

### Data Collection

#### Basic Sample Characteristics.

Data on age, gender, ethnicity, level of education, and employment status were collected using a modified version of the Medical Research Council socio-demographic schedule.^6,59^ DSM-IV diagnoses of psychotic disorder were determined based on structured examination of case records using the OPerational CRITeria (OPCRIT) system^60,61^ as part of the “Functional Enviromics” work package of EU-GEI.^6^ In the ARMS sample, current comorbid affective disorders were assessed with the SCID^56^ as part of the “G × E Prodrome” work package of EU-GEI.^6^ Data on medication use was collected using a medication checklist, which was completed based on close examination of clinical documentation, recording the use of all prescribed antipsychotic, antidepressant and other psychotropic medication.

#### ESM Measures.

Data on stress, negative affect, aberrant salience, threat anticipation, and psychotic experiences were collected using the ESM to allow for assessing moment-to-moment variation in these variables prospectively, in the real world and in real time, with high ecological validity. Specifically, we used a time-based design with stratified random sampling (ie, with ESM assessments scheduled at random within set blocks of time).^19,24,26,62,63^ While ESM data collection intense and resource heavy, previous research in samples of patients with psychotic disorder,^24,64^ ARMS individuals,^26^ and controls^24,26^ has demonstrated the feasibility, reliability, and validity of the assessment method.^19,63^ All participants were given an electronic momentary assessment technology device (the PsyMate).^65^ A detailed description of the ESM procedure and measures used^14,24,26,27,45,46,66–68^ is shown in table 1.

**Table 1.** ESM Procedure^a^ and Measures^b^ of Stress, Negative Affect, Aberrant Salience, Threat Anticipation, and Psychotic Experiences

Domain	^b^ESM Measure
Stress	Event-related, activity-related, and social stress were operationalized as minor disturbances and distinctive unpleasant events, activities, and social situations that occur in the natural flow of daily life based on previous ESM studies, in which good concurrent validity with other stress measures has been reported.^24,26^
Event	Event-related stress was measured with one item asking participants to rate the most important event since the last beep on a 7-point Likert scale ranging from “very unpleasant” (rating of −3) to “very pleasant” (rating of 3).^24^ We reversed the coding of this item in order for higher ratings to indicate higher levels of stress (with ratings of −3 (ie, “very unpleasant”) coded as 7 and ratings of 3 (ie, “very pleasant”) coded as 1).^24^
Activity	The mean score of 3 items (“I would prefer doing something else”, “This activity is difficult for me”, “This is a pleasant activity”(reversed)) rated on a 7-point Likert scale ranging from “not at all” (rating of 1) to “very much” (rating of 7) was used as activity-related stress scale.^24,26^
Social	The ESM social stress measure we used consisted of 2 items to assess moments where an individual’s current social environment induces minor stress in the natural flow of daily life (based on previous ESM studies^24,26^). Participants were first asked to indicate on a categorical item “Who am I with?” (partner, family, friends, colleagues, acquaintances, strangers, others, nobody) and then asked to rate their current social context on a 7-point Likert scale (ranging from “not at all” (rating of 1) to “very much” (rating of 7)) using the following 2 items: 1) “I would prefer to be alone [if with someone]/I would prefer to have company [if alone]”; 2) “I find being with these people pleasant [if with someone]/it pleasant to be alone [if alone]” The coding of item 2 was reversed and the mean score of these 2 items computed as a measure of minor social stress in daily life.^24,26^
Outsider status	Following ratings of current social context, participants were asked to rate one item (“I feel I am an outsider”) on a 7-point Likert scale (ranging from 1 [“not at all”] to 7 [“very much”]) to assess experiences of outsider status.
Area-related	Area-related stress was assessed by asking participants to rate one item “I find being in this neighbourhood unpleasant” on a 7-point Likert scale ranging from 1 (“not at all”) to 7 (“very much”).
Negative affect	We used a 5-item ESM measure for assessing negative affect. This measure asks participants to rate the extent to which they feel anxious, down, lonely, insecure, and annoyed at each entry point on a 7-point Likert scale ranging from 1 (“not at all”) to 7 (“very much”).^24^
Experiences of aberrant novelty and salience	A modified version of the 3-item ESM measure of aberrant salience by So^67^ was employed, asking participants to rate the following items on a 7-point Likert scale (ranging from 1 [“not at all”] to 7 [“very much”]): “Everything grabs my attention right now”, “Everything seems to have meaning right now”, and “I notice things that I haven’t noticed before.”^67^
Threat anticipation	Our ESM measure of threat anticipation was based on a self-report format used for assessing this mechanism in previous cross-sectional studies asking participants to rate the likelihood of negative events happening to them in the future.^14,45,46,68^ At each entry point, participants were asked to think of what might happen in the next few hours and to rate the item “I think that something unpleasant will happen” on a 7-point Likert scale (ranging from 1 [“not at all”] to 7 [“very much”]).
Psychotic experiences	The ESM psychosis measure was used to assess intensity of psychotic experiences. It consists of 7 items (eg, “I feel paranoid”, “I hear things that aren’t really there”, “My thoughts are influenced by others,” etc.) rated on a 7-point Likert scale (ranging from 1 [“not at all”] to 7 [“very much”]).^26,27^

^a^ *ESM procedure:* On each day over an assessment period of 6 consecutive days, the PsyMate emitted 10 “beep” signals at random moments within set blocks of time. During an initial briefing session, we trained participants in the use of the PsyMate by providing detailed technical instructions (eg, switching on/off, use of stylus for answering questions, etc.) and practising its usage by going through a practice questionnaire. In this session, participants were further given instructions about the ESM assessment and asked to stop their activity and respond to the above items each time the device emitted the beep signal as part of a more comprehensive diary questionnaire assessing thoughts, feelings, activities, behaviors, social situations, and neighbourhood surroundings in daily life. During the assessment period, which was selected to start at any day of the week at discretion of the participants (to optimize compliance and achieve sufficient spread of week and weekend days in our sample), the ESM questionnaire was available to participants for the duration of 10min after emission of the beep signal. Participants were contacted at least once during the assessment period to assess their adherence to instructions, identify any potential distress associated with the method, and help participants overcome any potential barriers for completing the questionnaire in order to maximise the number of observations per participant. At the end of the assessment period, participants’ reactivity to, and compliance with, the method was examined in a debriefing session. Participants were required to provide valid responses to at least one-third of the emitted beeps to be included in the analysis.^66^

### Statistical Analysis

We compared basic sample characteristics and ESM aggregate scores (ie, mean scores for each participant over the 6-day period) in FEP individuals, ARMS individuals, and controls using χ^2^-tests and linear regression as appropriate. ESM data have a multilevel structure, such that multiple observations (level-1) are nested within participants (level-2). Linear mixed models were therefore used to control for within-subject clustering of multiple observations using the “xtmixed” command in Stata 13.^69^ Maximum likelihood estimation of these models allows for the use of all available data under the relatively unrestrictive assumption that data is missing at random and if all variables associated with missing values are included in the model.^70,71^ First, we fitted separate models with each type of momentary stress as the independent variable and momentary negative affect as the outcome variable and, from these, generated fitted values (substituting maximum likelihood estimates for fixed effects and empirical Bayes predictions for random effects) for quantifying momentary stress sensitivity (ie, the association between stress and negative affect) for use in subsequent models. Second, we included variables associated with missing values (ie, age, group), adjusted these models for potential confounders (ie, gender, ethnicity, level of education, employment status), and added 2-way, stress × group interactions to test whether associations between stress and negative affect were stronger in FEP and ARMS individuals compared with controls. Third, models with psychological mechanisms (momentary event-related, activity-related, social, and area-related stress sensitivity, sensitivity to experiences of outsider status, aberrant salience, threat anticipation) as independent variables and momentary psychotic experiences as the outcome variable were fitted, while controlling for potential confounders and including variables associated with missing values in the model. We then added 2-way interaction terms for psychological mechanism × group to the adjusted main effects model and used likelihood ratio tests to evaluate improvement in model fit as well as the “lincom” command to compute linear combinations of coefficients for testing our hypotheses whether associations between psychological mechanisms and psychotic experiences were modified by group.

## Results

### Basic Sample Characteristics

A total of 165 participants (59 FEP, 51 ARMS, 55 controls) were assessed with the ESM during the study period. Of these, 150 participants (51 FEP, 46 ARMS individuals, 53 controls) completed ESM assessment (with ≥20 valid responses) and, therefore, a high proportion of those initially assessed were included in the analysis (ie, 90.9% of 165; supplementary table 2). There was only weak evidence that, compared with FEP individuals (86.4%), an (even) higher proportion of controls (96.4%) provided ≥20 valid responses (*P* = 0.179; supplementary table 2). The ARMS sample included 40 individuals recruited from OASIS and WLMHT, and 6 individuals from the community survey. Controls were on average older than FEP individuals and FEP older than ARMS individuals (table 2). The control group included slightly more women and individuals of White British ethnicity than the FEP group. FEP and ARMS individuals were more often unemployed and educated to school level than controls.

**Table 2.** Basic Sample Characteristics^a^

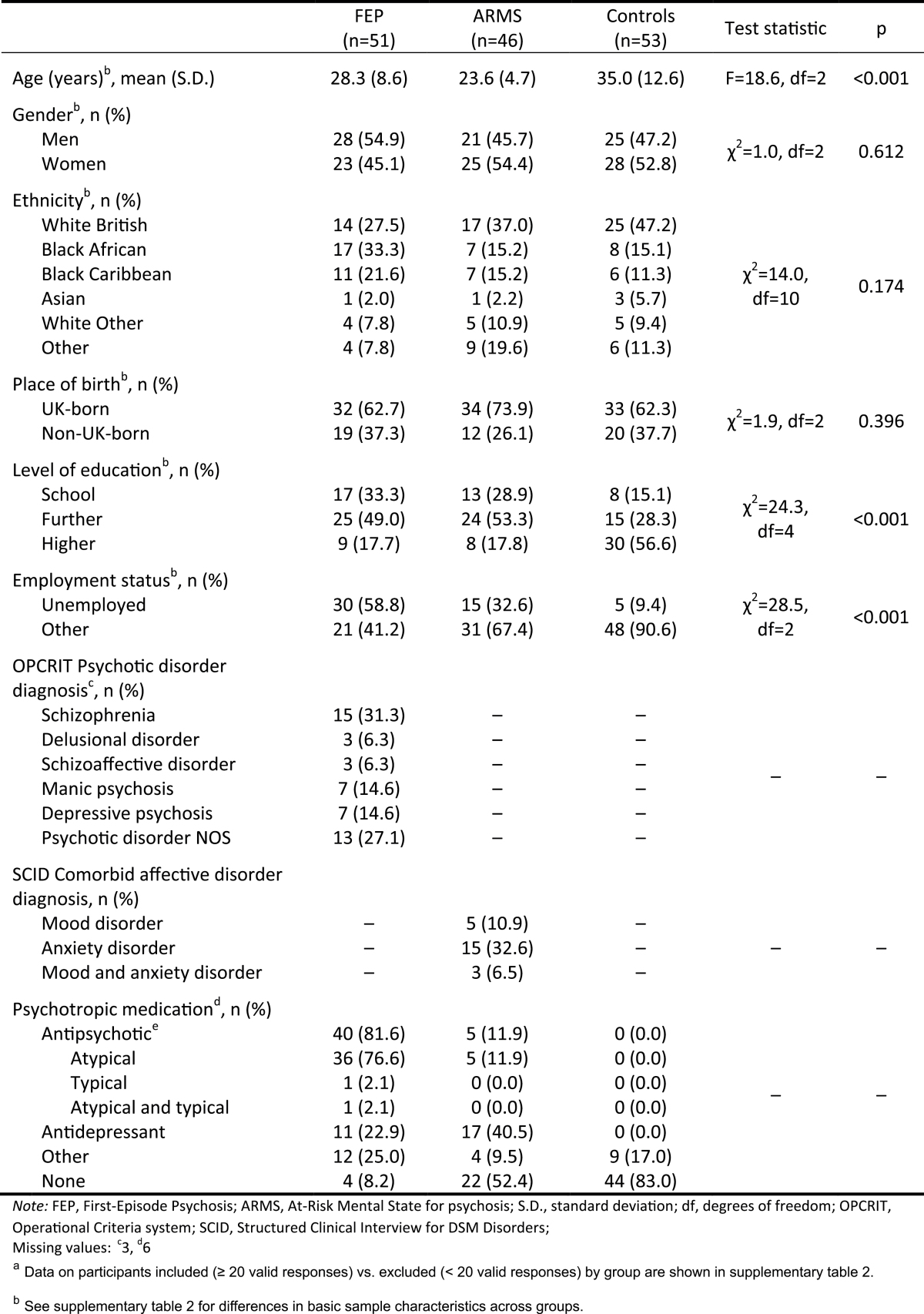

### Aggregate ESM scores in FEP, ARMS, and controls

Aggregate ESM scores in FEP, ARMS, and controls are shown in supplementary table 3. FEP and ARMS individuals experienced more event-related, activity-related, social, area-related, and outsider status-related stress as well as greater negative affect during the assessment period. Further, experiences of aberrant salience, enhanced threat anticipation, and psychotic experiences were more common in FEP and ARMS individuals than in controls.

### Momentary Stress Sensitivity in FEP, ARMS, and controls

Table 3 shows findings on momentary stress sensitivity (ie, the association between each type of momentary stress and negative affect) in FEP, ARMS, and controls. Within each group, each type of stress was associated with a small to moderate increase in negative affect (all *P* < 0.001). We also found evidence for interaction effects of stress × group on negative affect. This indicated that negative emotional reactions to event-related, activity-related, and outsider status-related stress were stronger in ARMS individuals compared with controls. Further, activity-related and outsider status-related stress sensitivity was elevated in FEP individuals compared with controls. However, there was no evidence of elevated area-related stress sensitivity in FEP and ARMS individuals compared with controls (*P* = 0.269).

**Table 3.** Momentary Stress Sensitivity, Characterized by Elevated Negative Affect in Response to Stress, by Group^a^

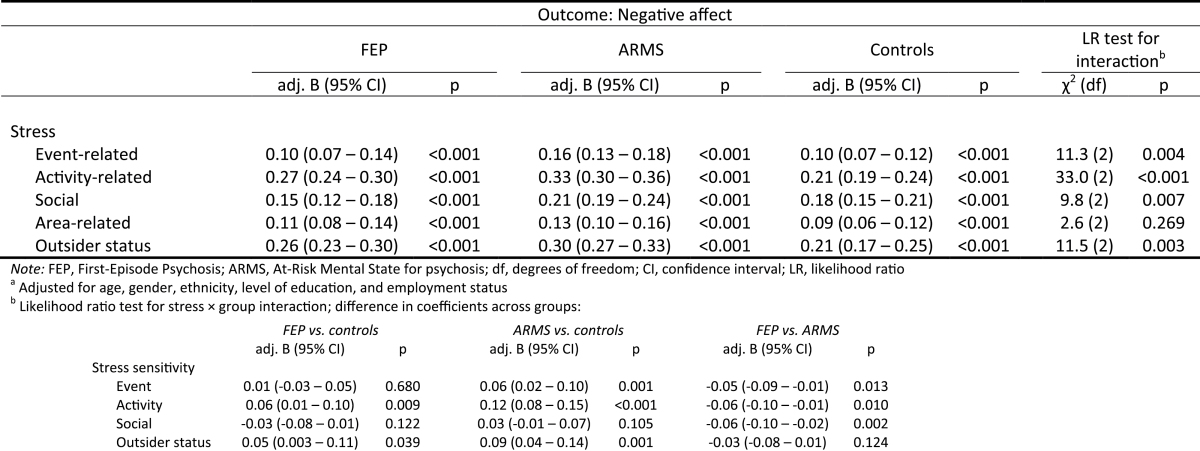

### Psychological Mechanisms and Momentary Psychotic Experiences by Group

Table 4 shows findings on the association between momentary stress sensitivity, aberrant salience, threat anticipation, and momentary psychotic experiences in FEP, ARMS, and controls. Within each group, there was strong evidence that elevated stress sensitivity, aberrant salience and enhanced threat anticipation were associated with an increased intensity of psychotic experiences (all *P* < 0.001). Further, the magnitude of these associations was modified by group as indicated by psychological mechanism × group interaction effects on psychotic experiences. The association between elevated event- (supplementary figure 1a), activity-, and area-related stress sensitivity, threat anticipation (supplementary figure 1b), and more intense psychotic experiences was moderately stronger in FEP individuals than in controls (all *P* < 0.024). Further, elevated activity-related stress sensitivity and aberrant salience (supplementary figure 1c) were associated with a greater increase in intensity of psychotic experiences in ARMS individuals than in controls (all *P* < 0.021). Also, there was some tentative evidence (*P* = 0.058) that elevated sensitivity to experiences of outsider status was associated with more intense psychotic experiences in ARMS individuals compared with controls. When comparing FEP with ARMS, elevated event- and area-related stress sensitivity as well as enhanced threat anticipation were associated with more intense psychotic experiences in FEP than in ARMS individuals (all *P* < 0.043), whereas experiences of aberrant salience related to more intense psychotic experiences in ARMS than in FEP individuals (*P* = 0.003). Finally, differences in the association between social stress sensitivity and psychotic experiences across groups fell short of statistical significance (*P* = 0.320).

**Table 4.** Momentary Stress Sensitivity, Aberrant Salience, Threat Anticipation, and Psychotic Experiences by Group^a^

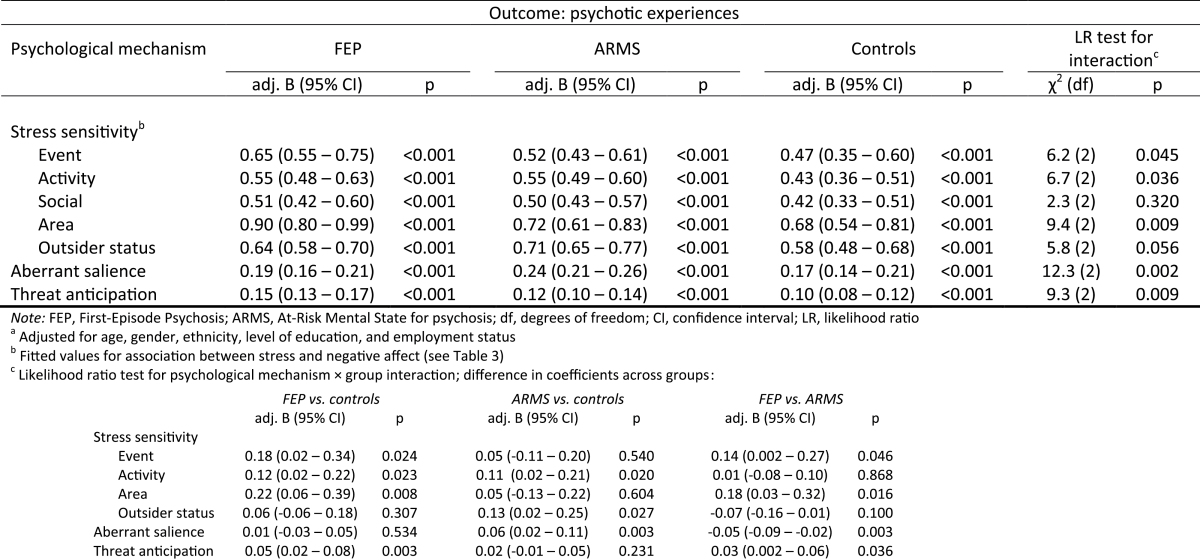

## Discussion

### Main Findings

This is the first study to investigate the role of elevated stress sensitivity, aberrant salience, and enhanced threat anticipation in the early stages of psychosis in a sample of FEP individuals, ARMS individuals, and controls in daily life. We found strong evidence in support of our first hypothesis that, within each group, elevated stress sensitivity, aberrant salience, and enhanced threat anticipation are associated with an increased intensity of psychotic experiences. Further, consistent with our second hypothesis, there was evidence that elevated event-, activity-, and area-related stress sensitivity and enhanced threat anticipation were associated with more intense psychotic experiences in FEP individuals compared with controls. Also, the increase in intensity of psychotic experiences associated with activity- and outsider status-related stress sensitivity as well as aberrant salience was greater in ARMS individuals compared with controls. However, there was no evidence of a stronger association between elevated social stress sensitivity and more intense psychotic experiences in FEP and ARMS individuals than in controls. Finally, our findings suggest that elevated event- and area-related stress sensitivity as well as enhanced threat anticipation were more relevant to the intensity of psychotic experiences in FEP than in ARMS individuals. By contrast, we found evidence that aberrant salience was more strongly associated with psychotic experiences in ARMS than in FEP individuals.

### Methodological Considerations

Several methodological considerations should be taken into account when interpreting findings from this study. First, while the ESM allowed psychological mechanisms and psychotic experiences to be assessed in the real world, with high ecological validity, all ESM ratings were based on subjective self-report. Our findings therefore still require triangulation with other psychological, biological, and socio-environmental measures. This may be particularly relevant for subjective ratings of area-related stress and outsider status, which presume specific socio-environmental exposures (eg, urban vs rural living, discrimination) impact on these mechanisms to increase intensity of psychotic experiences. Nevertheless, the ESM has been found to be a reliable and valid assessment method in ARMS and psychotic disorder in previous studies.^19,24,26,27^

Second, ESM data collection is time intense and may be associated with assessment burden for participants. While there was no difference in perceived assessment burden across groups, there was weak evidence that more controls than FEP individuals provided a sufficient number of valid responses to be included in the analysis. We therefore cannot rule out that, although unlikely, selection bias may have occurred as a result of this. Of those included in the analysis, on average, a higher number of valid responses was provided by controls than FEP and ARMS individuals (supplementary table 3), which may have reduced, to a degree, precision of effect estimates in the latter groups. Also, there was no formal requirement in our sampling strategy of a minimum number of valid responses per day. This may have led to sampling bias due to a lower number of responses on some days. However, through our extensive ESM recruitment, training, and adherence procedure (table 1), overall, there was a large proportion of participants with a sufficient number of valid responses to be included (supplementary table 2), a large number of participants providing responses on all 6 days (supplementary table 3), and, on average, a large number of valid responses in all 3 groups (supplementary table 3), which, coupled with maximum likelihood estimation (allowing for use of all available data),^70,71^ kept the potential impact of selection and sampling bias at a minimum.

Third, cross-sectional modeling of experience sampling data did not allow us to systematically examine temporal priority of putative psychological mechanisms over psychotic experiences. We therefore cannot rule out that the differences across groups may be explained by the different stages of early psychosis, with paranoid delusions driving enhanced threat anticipation in FEP individuals and attenuated psychotic symptoms leading to experiences of aberrant salience in ARMS individuals (not vice versa). Further, experiences of outsider status may have occurred as a consequence of stigma associated with, rather than adverse social environments prior to, psychotic disorder. Only a prospective design extending the age range into adolescence and following ARMS individuals over time would have allowed us to investigate causal criteria of psychological mechanisms underlying the occurrence and persistence of psychotic experiences as well as transition to psychotic disorder. We advanced, however, on previous research in restricting our sample of individuals with psychotic disorder to those with a first episode and, though (all but one) not antipsychotic-naïve, this sample allowed us to minimize the impact of illness chronicity, which may have affected findings from previous studies in enduring psychosis.^24,26^ Coupled with our ARMS sample without any prior treatment with an antipsychotic for a psychotic episode, this provided evidence on putative causal mechanisms prior to and at first onset of psychotic disorder. The slightly higher proportion of ethnic minority and unemployed individuals in the FEP group is consistent with, and may potentially be a reflection of, the higher incidence of psychosis among non-White British populations^29,72^ and the role of unemployment in FEP.^31,32^ While our analyses controlled for a range of confounders, we cannot rule out the possibility of unmeasured confounding by other important factors such as a higher socio-economic status of (the more highly educated) controls, which might have rendered this group more resilient and led to lower sensitivity to stress in this group.

Last, we recruited ARMS individuals from MHS and a community survey and presence of an ARMS was based on the CAARMS or SPI-A. While CAARMS and SPI-A have both been designed to determine presence of an ARMS, this may have resulted in heterogeneity in clinical characteristics in this sample.^4,52,73^ However, when we performed a sensitivity analysis to allow for comparison with previous studies in ARMS individuals from MHS^26^ and excluded ARMS individuals identified in the community survey, findings remained largely unchanged (supplementary table 4).

### Comparisons with Previous Research

Recent years have seen a move toward integrated models of psychosis.^9,74,75^ These models have posited that a number of psychological mechanisms contribute to the development of psychotic experiences,^7–11,44^ but there has been only a limited amount of research to inform our understanding of these mechanisms in individuals’ daily lives. While we found stress, negative affect, aberrant salience, threat anticipation, and psychotic experiences to be more common in FEP and ARMS individuals compared with controls, there was strong evidence that stress sensitivity, aberrant salience, and threat anticipation are important mechanisms underlying the development of more intense psychotic experiences in daily life across all 3 groups. This suggests these mechanisms are relevant across the different stages of early psychosis.

Echoing findings from Palmier-Claus et al,^26^ ARMS individuals reported greater activity-related and social stress sensitivity (characterized by stronger emotional reactions to minor activity-related and social stress) when compared with FEP individuals and controls. In contrast to this earlier study, we also found event-related stress sensitivity to be elevated in ARMS individuals. Consistent with Myin-Germeys et al’s^24^ findings in individuals with enduring psychosis, activity-related stress sensitivity was elevated in FEP individuals compared with controls, but, at variance with this study, no differences were observed across these 2 groups in event-related stress sensitivity. When we probed these findings further and moved beyond previous research^20,22,26,27^ to study the role of stress *sensitivity* in the development of psychotic experiences per se, we found event-, activity-, and area-related stress sensitivity to be more strongly associated with psychotic experiences in FEP individuals than in controls. Further, the association between event- and area-related stress sensitivity and psychotic experiences was even greater in FEP than in ARMS individuals, with some evidence of a dose-response gradient across the 3 groups. Put together, this tentatively suggests that, while individuals may be more sensitive to the effects of stress in the prodromal period when a considerable proportion experience comorbid anxiety and depression,^76^ this mechanism may be more relevant to increasing intensity of psychotic experiences at first onset of psychotic disorder. Viewed this way, this finding seems to parallel the increase in striatal dopamine synthesis capacity previously observed in ARMS individuals as they transition to psychotic disorder.^77^

Our finding that area-related stress sensitivity is associated with psychotic experiences adds to previous research suggesting stress sensitivity is a candidate mechanism underlying variation in rates of psychosis in terms of place.^78–83^ While previous research has reported neural social stress sensitivity is elevated in individuals exposed to urban environments,^78,83^ our findings suggest, for the first time, that momentary sensitivity to neighbourhoods subjectively appraised as stressful is associated with more intense psychotic experiences. Geographical momentary assessment studies that allow for real-time tracking and linkage of neighbourhood surroundings with subjective ratings of these^84,85^ are now needed to elucidate further the interplay of psychological mechanisms and area-level socio-environmental exposures. Similarly, the finding that elevated sensitivity to outsider status is associated with psychotic experiences, though in line with previous research,^34^ needs to be further validated in the context of socio-environmental factors that may increase sensitivity to this form of social stress.

This study extended beyond previous experimental research into the role of aberrant salience in psychosis^40,41^ by investigating moment-to-moment variation in putative mechanism in daily life. We found evidence that aberrantly salient experiences are more strongly associated with psychotic experiences in ARMS than in FEP individuals and controls, which may point toward aberrant salience playing a role well before the onset of psychotic disorder.^36^ Also, there was some evidence that, compared with controls, elevated sensitivity to outsider status was associated with more intense psychotic experiences in ARMS but not in FEP individuals. Both aberrant salience and experiences of outsider status have been closely linked to a sensitization of the dopaminergic system as an underlying biological mechanism.^34–38,42^ The weaker associations between these mechanisms and psychotic experiences in FEP than in ARMS individuals may reflect the effect of antipsychotic medication on elevated dopamine function in the former but not the latter group.^36,41^

Consistent with findings from a series of cross-sectional and experimental studies,^14,44–47^ we found evidence that enhanced threat anticipation is associated with more intense psychotic experiences in daily life. Given this association was stronger in FEP than in ARMS individuals and controls, this mechanism seems to impact on individuals to increase intensity of psychotic experiences in particular, as Bentall et al^44,45^ argued, in the final stage of developing clinical psychosis.

## Conclusions

Our findings suggest that stress sensitivity, aberrant salience, and threat anticipation are important psychological processes in the development of psychotic experiences across the continuum underlying the early stages of psychotic disorder. While experiences of aberrant salience and sensitivity to outsider status may be predominantly operating before the onset of psychosis and potentially reflect an underlying sensitization of the dopaminergic system, the impact of event- and area-related stress sensitivity as well as enhanced threat anticipation on psychotic experiences appears to increase as individuals transition from subclinical psychosis to the formation of a psychotic disorder. Our efforts should now focus on developing and evaluating ecological momentary interventions that directly modify these putative mechanisms to reduce intensity of psychotic experiences in daily life, with the goal of preventing onset and improving outcomes of psychosis.^86^

## Supplementary Material

Supplementary material is available at http://schizophreniabulletin.oxfordjournals.org.

## Funding

Postdoctoral Research Fellowship of the UK National Institute for Health Research (NIHR-PDF-201104065 to U.R.); Veni grant from the Netherlands Organisation for Scientific Research (451-13-022 to U.R.); Wellcome Trust (WT087417 to C.M.); National Institute for Health Research (NIHR) Biomedical Research Centre for Mental Health at South London and Maudsley NHS Foundation Trust and King’s College London; and this work is an approved add-on study of the “The European Network of National Networks studying Gene-Environment Interactions in Schizophrenia” (EU-GEI), which is supported by funding from the European Union (European Community’s Seventh Framework Program [HEALTH-F2-2009–241909; Project EU-GEI]).

## Supplementary Material

Supplementary Data
