# Supplementary material for: Stress Sensitivity, Aberrant Salience, and Threat Anticipation in Early Psychosis: An Experience Sampling Study
Source: Schizophr Bull. 2016 Feb 1;42(3):712–22. doi: 10.1093/schbul/sbv190 (PMC4838104; doi:10.1093/schbul/sbv190)
Supplement: Supplementary Data [file supp_42_3_712__index.html]

Stress Sensitivity, Aberrant Salience, and Threat Anticipation in Early Psychosis: An Experience Sampling Study — Stress Sensitivity, Aberrant Salience, and Threat Anticipation in Early Psychosis: An Experience Sampling Study — Supplementary Data 

# Stress Sensitivity, Aberrant Salience, and Threat Anticipation in Early Psychosis: An Experience Sampling Study

## Supplementary Data

Data files

- Supplementary Data - Supplementary Data
