# Supplementary material for: Stress Sensitivity, Aberrant Salience, and Threat Anticipation in Early Psychosis: An Experience Sampling Study
Source: Schizophr Bull. 2016 Feb 1;42(3):712–22. doi: 10.1093/schbul/sbv190 (PMC4838104; doi:10.1093/schbul/sbv190)
Supplement: Supplementary Data [file supp_sbv190_Supplementary_Tables___1st_revision_final.doc]

**Supplementary Table 1.** ARMS criteria based on the Comprehensive Assessment of At-Risk Mental States (CAARMS)5,6

| ARMS criteria |
| --- |
| 1. Schizotypal personality disorder plus a recent decline in function (defined as i) a 30% drop in the Social and Occupational Functioning Assessment Scale (SOFAS) score from premorbid level, sustained for 1 month, and occurring within past 12 months; or ii) a SOFAS score of 50 or less for past 12 months or longer) 2. First degree relative with psychosis plus a recent decline in function (see above) 3. ‘Attenuated’ positive psychotic symptoms 4. Brief psychotic episode of less than one week duration that resolves without antipsychotic medication |

*Note:* ARMS, At-Risk Mental State for psychosis

**Supplementary Table 2.** Aggregate ESM scores for stress, negative affect, outsider status, aberrant salience, threat anticipation and psychotic experiences in FEP, ARMS, and controlsa

|  | | FEP |  | ARMS |  | Controls |  | FEP vs. controls | |  | ARMS vs. controls | |
| --- | --- | --- | --- | --- | --- | --- | --- | --- | --- | --- | --- | --- |
|  | | Mean (S.D.) |  | Mean (S.D.) |  | Mean (S.D.) |  | B (95% CI) | p |  | B (95% CI) | p |
|  | |  |  |  |  |  |  |  |  |  |  |  |
| Stress | |  |  |  |  |  |  |  |  |  |  |  |
|  | Event | 3.70 (0.77) |  | 3.53 (0.74) |  | 3.24 (0.69) |  | 0.46 (0.37 – 0.55) | <0.001 |  | 0.29 (0.19 – 0.38) | <0.001 |
|  | Activity | 3.42 (0.90) |  | 3.15 (0.78) |  | 3.0 (0.50) |  | 0.43 (0.34 – 0.52) | <0.001 |  | 0.15 (0.07 – 0.24) | 0.001 |
|  | Social | 3.48 (0.89) |  | 2.91 (0.84) |  | 2.66 (0.72) |  | 0.83 (0.72 – 0.92) | <0.001 |  | 0.25 (0.15 – 0.34) | <0.001 |
|  | Area | 2.69 (1.49) |  | 2.92 (1.67) |  | 1.87 (0.89) |  | 0.82 (0.71 – 0.92) | <0.001 |  | 1.05 (0.94 – 1.15) | <0.001 |
|  | Outsider status | 3.07 (1.62) |  | 2.54 (1.53) |  | 1.54 (0.74) |  | 1.52 (1.42 – 1.62) | <0.001 |  | 0.99 (0.89 – 1.10) | <0.001 |
| Negative affect | | 3.04 (1.23) |  | 3.0 (1.08) |  | 1.91 (0.70) |  | 1.13 (1.05 – 1.21) | <0.001 |  | 1.10 (1.02 – 1.18) | <0.001 |
| Aberrant salience | | 2.87 (1.27) |  | 2.40 (1.13) |  | 2.19 (1.22) |  | 0.68 (0.59 – 0.77) | <0.001 |  | 0.21 (0.12 – 0.31) | <0.001 |
| Threat anticipation | | 2.62 (1.25) |  | 2.97 (1.33) |  | 1.87 (0.84) |  | 0.75 (0.65 – 0.85) | <0.001 |  | 1.11 (1.01 – 1.21) | <0.001 |
| Psychotic experiences | | 2.55 (1.27) |  | 2.40 (1.13) |  | 1.47 (0.59) |  | 1.08 (1.01 – 1.15) | <0.001 |  | 0.93 (0.86 – 1.01) | <0.001 |

*Note:* ESM, Experience Sampling Method; FEP, First-Episode Psychosis; ARMS, At-Risk Mental State for psychosis; S.D., standard deviation; CI, confidence interval

^a^ Compliance and perceived assessment burden by group:

|  | FEP | ARMS | Controls | FEP vs. controls | | ARMS vs. controls | | FEP vs. ARMS | |
| --- | --- | --- | --- | --- | --- | --- | --- | --- | --- |
|  |  |  |  | B (95% CI) | p | B (95% CI) | p | B (95% CI) | p |
| Compliance (number of valid responses) |  |  |  | -9.50 (-13.20 – -5.81) | <0.001 | -6.53 (-10.32 – -2.74) | 0.001 | -2.97 (-6.80 – 0.85) | 0.127 |
| Mean | 36.16 | 39.13 | 45.66 |  |  |  |  |  |  |
| S.D. | 10.10 | 10.31 | 8.16 |  |  |  |  |  |  |
| Range (Min – Max) | 37 (20 – 57) | 39 (21 – 60) | 35 (23 – 58) |  |  |  |  |  |  |
| Perceived assessment burden, mean (S.D.) | 3.72 (1.56) | 3.92 (1.56) | 3.73 (1.36) | 0.05 (-0.52 – 0.62) | 0.860 | 0.13 (-0.46 – 0.71) | 0.667 | -0.08 (-0.67 – 0.51) | 0.799 |
| Completed ESM assessment days, n(%) |  |  |  |  |  |  |  |  |  |
| 6 days | 50 (98.0) | 45 (97.8) | 53 (100.0) | – | – | – | – | – | – |
| Less than 6 days | 1 (2.0) | 1 (2.2) | 0 (0.0) | – | – | – | – | – | – |

**Supplementary Table 3.** Momentary stress sensitivity, outsider status, aberrant salience, threat anticipation, and psychotic experiences by groupa

|  | | Outcome: psychotic experiences | | | | | | | |  |  | |
| --- | --- | --- | --- | --- | --- | --- | --- | --- | --- | --- | --- | --- |
| Psychological mechanism | | FEP | |  | Restricted ARMS sample^b^ | |  | Controls | |  | LR test for interaction^c^ | |
|  | | adj. B (95% CI) | p |  | adj. B (95% CI) | p |  | adj. B (95% CI) | p |  | χ^2^ (df) | p |
|  | |  |  |  |  |  |  |  |  |  |  |  |
| Stress sensitivity^d^ | |  |  |  |  |  |  |  |  |  |  |  |
|  | Event | 0.65 (0.55 – 0.75) | <0.001 |  | 0.58 (0.48 – 0.68) | <0.001 |  | 0.47 (0.35 – 0.60) | <0.001 |  | 5.0 (2) | 0.082 |
|  | Activity | 0.55 (0.48 – 0.62) | <0.001 |  | 0.60 (0.53 – 0.66) | <0.001 |  | 0.43 (0.36 – 0.51) | <0.001 |  | 10.9 (2) | 0.004 |
|  | Social | 0.51 (0.42 – 0.60) | <0.001 |  | 0.55 (0.47 – 0.64) | <0.001 |  | 0.42 (0.33 – 0.51) | <0.001 |  | 4.3 (2) | 0.116 |
|  | Area-related | 0.90 (0.80 – 0.99) | <0.001 |  | 0.73 (0.62 – 0.84) | <0.001 |  | 0.68 (0.54 – 0.81) | <0.001 |  | 8.9 (2) | 0.012 |
|  | Outsider status | 0.64 (0.58 – 0.70) | <0.001 |  | 0.73 (0.67 – 0.79) | <0.001 |  | 0.58 (0.48 – 0.68) | <0.001 |  | 7.5 (2) | 0.024 |
| Aberrant salience | | 0.19 (0.16 – 0.21) | <0.001 |  | 0.26 (0.23 – 0.29) | <0.001 |  | 0.17 (0.14 – 0.21) | <0.001 |  | 20.9 (2) | <0.001 |
| Threat anticipation | | 0.15 (0.13 – 0.17) | <0.001 |  | 0.12 (0.10 – 0.14) | <0.001 |  | 0.10 (0.08 – 0.12) | <0.001 |  | 9.1 (2) | 0.011 |

*Note:* FEP, First-Episode Psychosis; ARMS, At-Risk Mental State for psychosis; df, degrees of freedom; CI, confidence interval; LR, likelihood ratio

^a^ Adjusted for age, gender, ethnicity, level of education, and employment status

^b^ Restricted ARMS sample (n=40) excluding ARMS individuals identified through screening of control sample (n=6)

^c^ Likelihood ratio test for psychological mechanism × group interaction

^d^ Fitted values for association between stress and negative affect (see Table 3)
